# App based education programme to reduce salt intake (AppSalt) in schoolchildren and their families in China: parallel, cluster randomised controlled trial

**DOI:** 10.1136/bmj-2021-066982

**Published:** 2022-02-10

**Authors:** Feng J He, Puhong Zhang, Rong Luo, Yuan Li, Yuewen Sun, Fengge Chen, Yuhong Zhao, Wei Zhao, Daoxi Li, Hang Chen, Tianyong Wu, Jianyun Yao, Changxing Lou, Siyuan Zhou, Le Dong, Yu Liu, Xian Li, Jing He, Changqiong Wang, Monique Tan, Jing Song, Graham A MacGregor

**Affiliations:** 1 Wolfson Institute of Population Health, Barts and London School of Medicine and Dentistry, Queen Mary University of London, London, UK; 2 George Institute for Global Health at Peking University Health Science Centre, China; 3 Faculty of Medicine, University of New South Wales, Sydney, NSW, Australia; 4 Shijiazhuang Centre for Disease Control and Prevention, Shijiazhuang, Hebei Province, China; 5 Chang’an Centre for Disease Control and Prevention, Shijiazhuang, Hebei Province, China; 6 Luzhou Centre for Disease Control and Prevention, Luzhou, Sichuan Province, China; 7 Yueyang Centre for Disease Control and Prevention, Yueyang, Hunan Province, China; 8 School of Computer Science and Engineering, Beihang University, Beijing, China

## Abstract

**Objective:**

To determine whether a smartphone application based education programme can lower salt intake in schoolchildren and their families.

**Design:**

Parallel, cluster randomised controlled trial, with schools randomly assigned to either intervention or control group (1:1).

**Setting:**

54 primary schools from three provinces in northern, central, and southern China, from 15 September 2018 to 27 December 2019.

**Participants:**

592 children (308 (52.0%) boys; mean age 8.58 (standard deviation 0.41) years) in grade 3 of primary school (about 11 children per school) and 1184 adult family members (551 (46.5%) men; mean age 45.80 (12.87) years).

**Intervention:**

Children in the intervention group were taught, with support of the app, about salt reduction and assigned homework to encourage their families to participate in activities to reduce salt consumption.

**Main outcome measures:**

Primary outcome was the difference in salt intake change (measured by 24 hour urinary sodium excretion) at 12 month follow-up, between the intervention and control groups.

**Results:**

After baseline assessment, 297 children and 594 adult family members (from 27 schools) were allocated to the intervention group, and 295 children and 590 adult family members (from 27 schools) were allocated to the control group. During the trial, 27 (4.6%) children and 112 (9.5%) adults were lost to follow-up, owing to children having moved to another school or adults unable to attend follow-up assessments. The remaining 287 children and 546 adults (from 27 schools) in the intervention group and 278 children and 526 adults (from 27 schools) in the control group completed the 12 month follow-up assessment. Mean salt intake at baseline was 5.5 g/day (standard deviation 1.9) in children and 10.0 g/day (3.5) in adults in the intervention group, and 5.6 g/day (2.1) in children and 10.0 g/day (3.6) in adults in the control group. During the study, salt intake of the children increased in both intervention and control groups but to a lesser extent in the intervention group (mean effect of intervention after adjusting for confounding factors −0.25 g/day, 95% confidence interval −0.61 to 0.12, P=0.18). In adults, salt intake decreased in both intervention and control groups but to a greater extent in the intervention group (mean effect −0.82 g/day, −1.24 to −0.40, P<0.001). The mean effect on systolic blood pressure was −0.76 mm Hg (−2.37 to 0.86, P=0.36) in children and −1.64 mm Hg (−3.01 to −0.27, P=0.02) in adults.

**Conclusions:**

The app based education programme delivered through primary school, using a child-to-parent approach, was effective in lowering salt intake and systolic blood pressure in adults, but the effects were not significant in children. Although this novel approach could potentially be scaled up to larger populations, the programme needs further strengthening to reduce salt intake across the whole population, including schoolchildren.

**Trial registration:**

Chinese Clinical Trial Registry ChiCTR1800017553.

## Introduction

High salt intake is a major dietary risk factor, responsible for about 1.8 million deaths and 44 million disability adjusted life years worldwide in 2019.^1^ Systematic reviews and meta-analyses have shown that salt reduction lowers blood pressure^2^ ^3^ and reduces the risk of cardiovascular disease.^4^ ^5^ ^6^ Many health organisations including the World Health Organization have recommended a reduction in salt intake across the whole population.^6^ ^7^ ^8^ Several developed countries (eg, Finland and the UK) have successfully reduced salt intake, leading to reductions in population blood pressure and cardiovascular disease mortality.^9^ Developing countries, however, are lagging behind, despite more than 80% of the world’s salt related disease burden occurring in these countries.^10^

China is the largest developing country with a fifth of the world population. Salt intake in China is among the highest in the world.^11^ ^12^ Unlike other developed countries where about 80% of the salt is added by the food industry to processed, restaurant, and fast foods and snacks, in China about 80% of the salt is added by the consumers during cooking.^13^ It is very difficult to reduce salt intake in such settings owing to the challenge of changing individuals’ dietary behaviours. Our previous study in northern China has shown that a school based education programme (School-EduSalt) is effective in lowering salt intake in both children and their family members over an intervention period of one school term (about 3.5 months).^14^ Further innovative approaches would be needed to sustain salt reduction, because the school curriculum is overcrowded in China^15^ and lessons in salt reduction education would be unlikely to continue in schools over time despite being feasible in one school term.

Mobile technology is being increasingly integrated into health education.^16^ Game based interventions have the potential to change behaviours owing to the popularity of gaming.^17^ In China, more than 800 million people used the internet, of whom 98.3% had internet access through mobile devices in June 2018.^18^ We previously developed a smartphone application (KnowSalt) that could be used by individuals to estimate their salt intake and the major sources of salt in their diet.^19^

For this present study, building on School-EduSalt and KnowSalt, we have developed a new smartphone application focusing on salt reduction (AppSalt) through functional modules of education, evaluation, target setting and monitoring, decision support and management.^20^ To test whether the app based programme could achieve and sustain a lower salt intake, we carried out a cluster randomised trial.

## Methods

A detailed description of the methods has been published elsewhere.^20^

### Study design and participants

The parallel, cluster randomised controlled trial was carried out between 15 September 2018 and 27 December 2019 in primary schools from three cities: Shijiazhuang in Hebei province, Luzhou in Sichuan province, and Yueyang in Hunan province. These sites are located in northern, central, and southern China, respectively. From each school, we recruited one class in grade 3 (age 8-9 years). From each class, we randomly selected 11 children and 22 adult family members (that is, one child and two adults per family) for outcome assessments. Inclusion criteria were:

- Children and their adult family members who ate homemade meals at least four times per week
- One adult family member had to have a mobile device with internet access
- If more than two adults in one family agreed to take part in the assessments, we selected two adults in the order of grandparents, parents, and uncles and aunts
- Participants had been local residents for more than six months.

Individuals who could not or refused to collect 24 hour urine samples were excluded from the outcome assessments.

### Randomisation and masking

A computer generated, central randomisation system (at the George Institute for Global Health at Peking University Health Science Centre, China) allocated schools (clusters) to either intervention or control group (1:1), stratified by the location of schools (that is, southern, central, or northern China). Randomisation took place after baseline data collection. Therefore, the participants, schoolteachers, and local investigators who undertook recruitment and data collection were unaware of the allocation up until the start of the intervention.

### Procedures

The main components of the intervention were salt reduction education and monitoring via the app based platform, complemented by the creation of supportive environments (eg, salt awareness posters put up in classrooms, campuses, and canteens) and offline activities that included art and knowledge competitions, and seminars for both children and adults organised by teachers, coinciding with the schools’ routine parent meetings. A detailed description of the intervention activities is provided in supplement 1.

The app based platform comprised the AppSalt app installed on the parents’ smartphones (family end) and a WeChat mini app installed on the teachers’ smartphones (teacher end).^20^ AppSalt contains nine lessons on salt reduction education (eg, on the harmful effects of salt on health and the methods of reducing salt intake) and 12 health education lessons as normally included in the curriculum that did not contain salt related material, such as flu prevention. All lessons were 10 minutes each, spanning over the 12 month intervention period (that is, two school terms). At the end of each lesson, families were asked to complete an online question and answer session and a practical session (eg, preparing foods with less salt at home or selecting lower salt products when shopping). Only adults were authorised to operate the app. Children’s tasks were to ensure that their parents (or grandparents) completed the lessons together with them and to involve the whole family in salt reduction activities.

Salt intake was monitored by a salt estimation method over seven days.^19^ Participants were asked to complete a seven day diary on salt intake and recorded the data in the app. This process included weighing salt and other condiments (eg, soy sauce) used in home cooking, estimating the consumption of processed food high in salt, and recording the frequency of eating out during the seven days. After completing this diary, the app calculated the average salt consumption using an embedded algorithm and generated a salt reduction action plan for each family member according to their salt intake and major sources of salt in their diet. All families were asked to complete this procedure at baseline and every 3-4 months thereafter. The information obtained could help each family member set a lower salt intake target and salt reduction targets for the top three contributors—for example, reducing salt used in cooking by 50%. At the same time, the data shown on the app could help individuals monitor their progress on achieving lower salt intake.

Trained teachers provided the families with guidance on AppSalt use and assigned and assessed children’s homework. Based on the progress report from the WeChat mini app, the teachers would remind the children to complete their homework—that is, help their families complete the AppSalt procedures and instructing family members to achieve their salt reduction target. In the intervention group, all children and their families in the class received the intervention, despite only 11 children and their families being randomly selected for outcome assessment. Children in the control group did not receive the intervention.

### Outcomes

The primary outcome was the difference between the intervention and control groups in salt intake change, as measured by 24 hour urinary sodium excretion from baseline to the end of the trial (12 months after randomisation) for children and for adults. The secondary outcome was the difference between intervention and control groups in the change of systolic blood pressure in adults.

All outcome measurements were carried out at baseline and at the end of the trial in an identical way for all participants, irrespective of group assignment. Two consecutive 24 hour urine collections were made at each time point, using the stringent protocol that we developed in the School-EduSalt study.^14^ Urine collections were also supported by an electronic data collection system, which helped standardise the collection procedure.^21^ The urine samples were measured for volume, and sodium, potassium, and creatinine levels. We used an ion selective electrode method to measure sodium and potassium, and an enzymatic method to measure creatinine. Biochemists who measured the urinary electrolyte levels were not aware of participants’ group allocation.

Trained researchers measured blood pressure by using a validated automatic machine with the appropriate size of cuff. Three readings were taken in the right arm at 1-2 minute intervals while participants were in the sitting position after a 10 minute rest. Body weight and height were measured following a standardised protocol.

### Statistical analysis

Based on the findings from School-EduSalt,^14^ and assuming a standard deviation of 24 hour urinary sodium of 85 mmol and an intraclass correlation coefficient of 0.05, we estimated that a sample size of 594 children from 54 schools would provide a power of 80% (α=0.05) to detect a difference in mean 24 hour urinary sodium ≥26 mmol (1.5 g/day of salt) between the two randomised groups, allowing for a 15% dropout rate of participants. We planned to recruit two adults per family; therefore, 1188 adults would be recruited for outcome assessments.

We excluded 24 hour urine collections from the main analysis if the collection time was less than 20 hours or more than 28 hours, or if one or more urine samples were missed or spilt. If the duration of collection was not exactly 24 hours but within 20-28 hours, the 24 hour urine volume was calculated as: urine volume (mL per 24 hours) = (urine volume (mL) ÷ duration of urine collection (hours)) × 24 hours. We calculated 24 hour urinary sodium, potassium, and creatinine excretions by multiplying these urinary concentrations with 24 hour urine volume. Urine samples were also considered incomplete and excluded from the main analysis if 24 hour urine volume was <500 mL in adults; 24 hour urinary creatinine was <4.0 mmol or <6.0 mmol in women or men, respectively; 24 hour urine volume was <300 mL in children; or 24 hour urinary creatinine was lower than the 5th centile (<2.33 mmol for girls and <3.11 mmol for boys). The average of the two 24 hour urinary measurements at each time point was used in the analysis. If one of these two 24 hour urine collections was incomplete, we used the urinary values from the complete 24 hour urine rather than the average of the two. For blood pressure, we used the average of the last two of the three readings in the analysis.

Data analysis was performed according to the intention-to-treat approach—that is, schools and participants who completed the baseline assessment were analysed according to their randomly assigned group. We tested the effects of the intervention on outcomes by using mixed linear models taking into account the hierarchical structure of data with two measurements (baseline and 12 month) for each participant, participants nested in each family, and family nested in each school. The models included random intercept and fixed effect variables (eg, time, group, time×group interaction, and covariates). The time×group interaction term indicated the differential change by group from baseline to the end of the trial (that is, 12 months after randomisation). We adjusted for the stratification variable at randomisation (study site) and potential confounding variables including age, sex, body mass index (body weight in children), outdoor temperature, and highest education level in the families (primary education or less, secondary education, high school education, and university or college education). All analyses for blood pressure were further adjusted for physical activity (coded as “yes” if the participant reported engaging in moderate physical activity for at least 30 minutes and at least three times a week; otherwise coded as “no”). For adults, additional adjustment was made for alcohol consumption (coded as “non-drinker” if the participant answered “no” to the question “do you drink alcohol”; “occasional drinker” if the participant answered “sometimes”; and “regular drinker” if the participant answered “always” or “addicted”).

We performed two sensitivity analyses to examine the robustness of the intervention effect on the primary outcome. The first analysis was based on an intention-to-treat approach that included possibly incomplete 24 hour urine collections, assessed by 24 hour urinary volume and creatinine excretion. The second analysis included only participants who completed both baseline and end trial assessments, and who also had complete 24 hour urine collections at both collection time points.

We also carried out a post hoc subgroup analysis by sex, study site, and highest education in the family for children. For adults, the post hoc subgroup analysis was by age group, sex, study site, education attainment, blood pressure status (hypertension was defined as systolic blood pressure ≥140 mm Hg or diastolic blood pressure ≥90 mm Hg or self-reported hypertension), and adults’ relationship with the children (that is, parents, grandparents, or other family members). We did subgroup analysis by adding the subgroup variable; its two way interaction with time and group, respectively (subgroup variable×group, subgroup variable×time); and its three way interaction with time and group (subgroup variable×time×group). Analysis of variance with type III sum of squares was used to test the fixed effect of the three way interaction, which was reported as the P value for difference in intervention effect across subgroups. Results were reported as means, standard deviations, and 95% confidence intervals where appropriate. All analyses were two sided and P<0.05 was considered significant. The statistical analyses were performed using SAS software, version 9.3 (SAS Institute).

### Patient and public involvement

Schoolteachers, head teachers, and children’s parents were involved in the design and conduct of this study. At the protocol development stage, meetings were held to gain their opinion on the content and function of the app based intervention, and the feasibility of conducting the study in school settings. In addition, experienced primary schoolteachers helped us to develop the education courses and translate the education materials that were produced by the research team into language suitable for 8-9 year old children. During the study, schoolteachers assigned and assessed children’s homework related to the education programme. Teachers and head teachers were informed of study progress by monthly communication via newsletters, WeChat, and website updates. Once the study is published, teachers, head teachers, parents, and participants will be informed of the results through the same communication channels.

## Results

**Fig 1:**
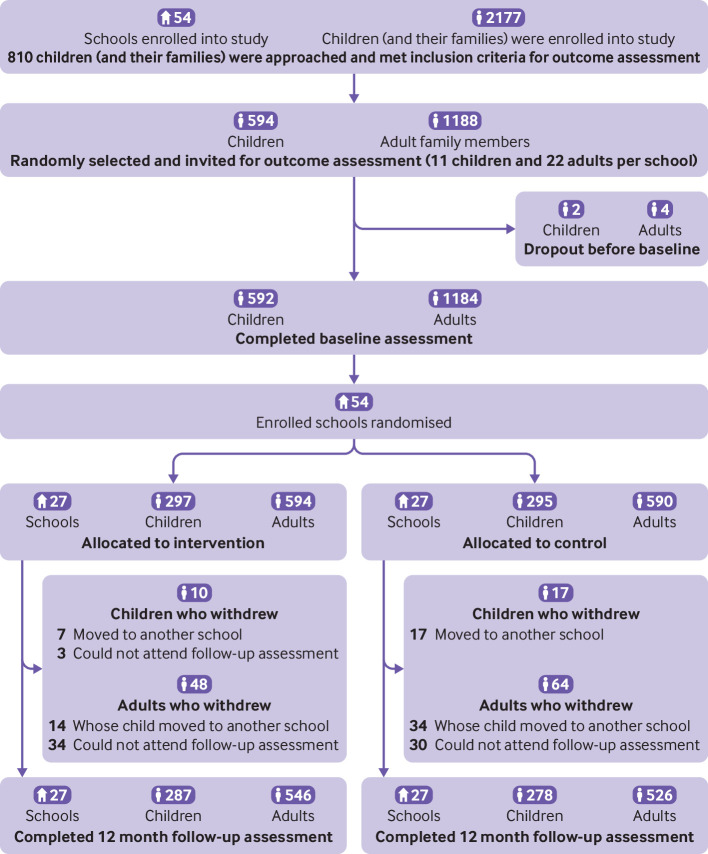
Trial profile of app based education programme to reduce salt intake in schoolchildren and their families

We recruited 54 schools (18 schools in each of the three sites, one class from each school) with 2177 children into the study. Among these children, 810 were approached by schoolteachers, and met the inclusion criteria for outcome assessments. A total of 594 children were randomly selected and invited to take part in the outcome evaluation (fig 1). We also invited 1188 adults from these children’s families, to take part in the outcome measurements. Among those invited, 592 children and 1184 adults completed the baseline assessments. During the trial, 27 (4.6%) children and 112 (9.5%) adults were lost to follow-up, owing to children having moved to another school or adults unable to attend follow-up assessments (fig 1). No significant difference was seen between participants who were lost to follow-up and those who completed the study in age, salt intake, body mass index, or blood pressure at baseline for children or adults (data not shown). Baseline characteristics of the study participants were generally similar between the two randomised groups (table 1) except for adults’ mean age, which was slightly higher in the intervention group (46.73 years (standard deviation 13.06) *v* 44.86 (12.61) years).

**Table 1:** Baseline characteristics. SD=standard deviation

Characteristic	Control	Intervention	Total
**Cluster level**			
No (%) of schools, by location			
Shijiazhuang	9/27 (33.3)	9/27 (33.3)	18/54 (33.3)
Yueyang	9/27 (33.3)	9/27 (33.3)	18/54 (33.3)
Luzhou	9/27 (33.3)	9/27 (33.3)	18/54 (33.3)
No (%) of families	295 (100.0)	297 (100.0)	592 (100.0)
Outdoor temperature (°C)	19.58 (4.81)	18.82 (5.02)	19.20 (4.93)
**Individual level**			
Total No (%) of participants	885 (100.0)	891 (100.0)	1776 (100.0)
**Children level**			
No (%) of boys/total	156 (52.9)/295	152 (51.2)/297	308 (52.0)/592
Mean (SD) age (years)	8.58 (0.47)	8.59 (0.34)	8.58 (0.41)
Mean (SD) weight (kg)	29.82 (6.93)	30.48 (7.47)	30.15 (7.21)
Mean (SD) body mass index	17.10 (2.89)	17.37 (3.10)	17.23 (3.00)
Physical activity (No (%)/total)			
No	151 (51.2)/295	142 (47.8)/297	293 (49.5)/592
Yes	144 (48.8)/295	155 (52.2)/297	299 (50.5)/592
Highest education in the family (No (%)/total)			
≤Primary education	25 (8.5)/295	26 (8.8)/297	51 (8.6)/592
Secondary education	55 (18.6)/295	72 (24.2)/297	127 (21.5)/592
High school education	100 (33.9)/295	93 (31.3)/297	193 (32.6)/592
University or college education	115 (39.0)/295	106 (35.7)/297	221 (37.3)/592
**Adult level**			
No (%) of men/total	279 (47.3)/590	272 (45.8)/594	551 (46.5)/1184
Relationship with children			
Parents	194 (32.9)/590	180 (30.3)/594	374 (31.6)/1184
Grandparents	260 (44.1)/590	251 (42.3)/594	511 (43.2)/1184
Other family members	136 (23.1)/590	163 (27.4)/594	299 (25.3)/1184
Mean (SD) age (years)	44.86 (12.61)	46.73 (13.06)	45.80 (12.87)
Mean (SD) weight (kg)	65.52 (12.68)	65.30 (12.60)	65.41 (12.63)
Mean (SD) body mass index	25.06 (3.63)	25.06 (3.78)	25.06 (3.70)
Physical activity (No (%)/total)			
No	399 (67.6)/590	405 (68.2)/594	804 (67.9)/1184
Yes	191 (32.4)/590	189 (31.8)/594	380 (32.1)/1184
Highest education (No (%)/total)			
≤Primary education	106 (18.0)/590	117 (19.7)/594	223 (18.8)/1184
Secondary education	140 (23.7)/590	161 (27.1)/594	301 (25.4)/1184
High school education	169 (28.6)/590	163 (27.4)/594	332 (28.0)/1184
University or college education	175 (29.7)/590	153 (25.8)/594	328 (27.7)/1184
Alcohol drinkers (No (%)/total)			
Non-drinkers	335 (56.8)/590	329 (55.4)/594	664 (56.1)/1184
Occasional drinkers	205 (34.7)/590	220 (37.0)/594	425 (35.9)/1184
Regular drinkers	50 (8.5)/590	45 (7.6)/594	95 (8.0)/1184
Self-reported hypertension (No (%))	69 (11.7)	71 (12.0)	140 (11.8)
Blood pressure treatment in adults with hypertension (No (%))	49 (71.0)	53 (74.6)	102 (72.9)

Of 297 families in the intervention group, 253 (85%) completed all of the nine app based salt reduction education lessons, and 237 (80%) completed the procedure for monitoring salt intake (that is, estimated salt intake over seven days) four or more times during the 12 month intervention period. Participation rates for the four offline competitions among children ranged from 72% (215/297) for the children’s art contest to 99% (293/297) for the salt reduction knowledge competition. Supplement figure S3 shows the changes in the average salt intake estimated by the app. According to these self-reported data, the estimated salt intake had a gradual and sustained reduction during the entire intervention duration of 12 months, for both children and adults.

Table 2 shows the results for salt intake (as measured by 24 hour urinary sodium excretion), other 24 hour urinary measurements, and blood pressure. In children, the mean baseline salt intake was 5.6 (standard deviation 2.1) g/day in the control group and 5.5 (1.9) g/day in the intervention group. During the 12 month study, salt intake increased in both groups, but to a lesser extent in the intervention group (table 2). The mean effect comparing the intervention group with the control group was −0.33 g/day (95% confidence interval −0.69 to 0.03). Blood pressure showed an increase from baseline to the end of the trial in both groups, and the mean effect comparing the intervention group with the control group was −1.19 mm Hg (−2.76 to 0.38) for systolic blood pressure and −1.28 mm Hg (−2.64 to 0.09) for diastolic blood pressure (table 2).

**Table 2:** Salt intake (as calculated from 24 hour urinary sodium excretion), other 24 hour urinary measurements, and blood pressure based on intention-to-treat analysis

	Control					Intervention					Difference (95% CI) in change (intervention *v* control)†, P value			Adjusted difference (95% CI) in change†‡ (intervention *v* control), P value	
	No of participants	Baseline mean (SD)	12 month mean (SD)	Change* (95% CI)		No of participants	Baseline mean (SD)	12 month mean (SD)	Change* (95% CI)						
**Children**															
Salt intake (g/day)	294	5.6 (2.1)	6.1 (2.3)	0.52 (0.26 to 0.78)		296	5.5 (1.9)	5.7 (2.3)	0.19 (−0.07 to 0.44)		−0.33 (−0.69 to 0.03)	0.07		−0.25 (−0.61 to 0.12)	0.18
Systolic blood pressure (mm Hg)	295	92.5 (9.8)	95.3 (8.7)	2.65 (1.53 to 3.77)		297	92.8 (9.7)	94.3 (9.9)	1.46 (0.36 to 2.56)		−1.19 (−2.76 to 0.38)	0.14		−0.76 (−2.37 to 0.86)	0.36
Diastolic blood pressure (mm Hg)	295	64.0 (8.2)	65.5 (7.4)	1.40 (0.43 to 2.37)		297	64.3 (8.5)	64.5 (7.8)	0.12 (−0.83 to 1.08)		−1.28 (−2.64 to 0.09)	0.07		−1.05 (−2.45 to 0.34)	0.14
Urinary sodium (mmol/24 hours)	294	95.3 (35.0)	104.3 (39.0)	8.88 (4.49 to 13.27)		296	94.1 (33.2)	96.9 (38.6)	3.16 (−1.16 to 7.48)		−5.72 (−11.88 to 0.44)	0.07		−4.23 (−10.46 to 2.00)	0.18
Urinary potassium (mmol/24 hours)	294	24.5 (8.0)	25.0 (8.3)	0.55 (−0.62 to 1.72)		296	24.3 (7.9)	25.1 (8.5)	0.75 (−0.41 to 1.90)		0.20 (−1.44 to 1.84)	0.81		0.29 (−1.39 to 1.98)	0.73
Sodium-to- potassium ratio	294	4.2 (1.8)	4.5 (2.0)	0.27 (0.02 to 0.53)		296	4.2 (1.7)	4.3 (2.3)	0.10 (−0.15 to 0.35)		−0.17 (−0.53 to 0.19)	0.35		−0.10 (−0.46 to 0.27)	0.61
Urinary creatinine (mmol/24 hours)	294	4.9 (1.3)	5.3 (1.2)	0.31 (0.19 to 0.44)		296	4.8 (1.2)	5.2 (1.3)	0.45 (0.33 to 0.58)		0.14 (−0.04 to 0.32)	0.13		0.14 (−0.04 to 0.33)	0.13
Urine volume (mL/24 hours)	294	812.5 (287.6)	895.5 (329.7)	79.05 (38.58 to 119.52)		296	877.4 (303.7)	926.8 (355.1)	47.04 (7.23 to 86.86)		−32.01 (−88.78 to 24.77)	0.27		−16.86 (−75.00 to 41.28)	0.57
**Adults**															
Salt intake (g/day)	587	10.0 (3.6)	9.8 (3.9)	−0.30 (−0.59 to −0.01)		593	10.0 (3.5)	8.9 (3.2)	−1.18 (−1.47 to −0.89)		−0.88 (−1.29 to −0.47)	<0.001		−0.82 (−1.24 to −0.40)	<0.001
Systolic blood pressure (mm Hg)	590	118.9 (16.8)	118.5 (17.5)	−0.32 (−1.29 to 0.65)		594	118.7 (17.6)	115.7 (16.5)	−2.86 (−3.81 to −1.90)		−2.53 (−3.90 to −1.17)	<0.001		−1.64 (−3.01 to −0.27)	0.02
Diastolic blood pressure (mm Hg)	590	77.5 (10.2)	76.6 (11.0)	−0.82 (−1.50 to −0.14)		594	76.9 (10.6)	74.7 (9.9)	−2.01 (−2.68 to −1.34)		−1.19 (−2.15 to −0.23)	0.02		−0.48 (−1.44 to 0.48)	0.33
Urinary sodium (mmol/24 hours)	587	171.6 (62.1)	167.6 (66.1)	−5.17 (−10.15 to −0.18)		593	171.7 (60.3)	151.6 (54.2)	−20.17 (−25.05 to −15.28)		−15.00 (−21.98 to −8.03)	<0.001		−14.02 (−21.20 to −6.83)	<0.001
Urinary potassium (mmol/24 hours)	587	39.9 (13.8)	39.6 (13.4)	−0.52 (−1.68 to 0.64)		593	39.4 (13.2)	39.3 (13.6)	−0.38 (−1.52 to 0.76)		0.14 (−1.49 to 1.76)	0.87		0.15 (−1.53 to 1.83)	0.86
Sodium-to- potassium ratio	587	4.6 (1.8)	4.5 (1.8)	−0.10 (−0.25 to 0.05)		593	4.6 (1.7)	4.2 (1.8)	−0.40 (−0.54 to −0.25)		−0.30 (−0.50 to −0.09)	0.005		−0.27 (−0.48 to −0.06)	0.01
Urinary creatinine (mmol/24 hours)	587	11.0 (3.2)	10.4 (3.0)	−0.57 (−0.73 to −0.41)		593	10.7 (3.1)	10.3 (3.0)	−0.48 (−0.64 to −0.32)		0.09 (−0.14 to 0.31)	0.45		0.05 (−0.18 to 0.28)	0.66
Urine volume (mL/24 hours)	587	1529.6 (593.9)	1491.6 (536.2)	−48.55 (−93.62 to −3.49)		593	1598.9 (582.1)	1556.3 (530.5)	−54.27 (−98.46 to −10.08)		−5.72 (−68.84 to 57.40)	0.86		36.80 (−27.48 to 101.08)	0.26

SD=standard deviation.

* Comparison of the means between baseline and 12 month follow-up. Positive values=increases from baseline to 12 month follow-up; negative values=reductions from baseline to 12-month follow-up.

† Comparison between intervention and control groups in the changes from baseline to 12 month follow-up. Positive values=the intervention group had a greater increase or less decrease from baseline to 12 month follow-up than the control group; negative values=the intervention group has a greater decrease or smaller increase from baseline to 12 month follow-up than the control group.

‡ Adjusted for age, sex, body mass index (body weight in children instead), outdoor temperature, study site, highest education level in the family; blood pressure values were further adjusted for physical activity and alcohol consumption in adults only.

In adults, the mean baseline salt intake was 10.0 (3.6) g/day in the control group and 10.0 (3.5) g/day in the intervention group. During the study, salt intake decreased in both groups, but to a greater extent in the intervention group, and the mean effect was −0.88 g/day (−1.29 to −0.47). Blood pressure decreased in both groups, but the extent of the blood pressure fall was greater in the intervention group and the mean effect was −2.53 mm Hg (−3.90 to −1.17) for systolic blood pressure and −1.19 mm Hg (−2.15 to −0.23) for diastolic blood pressure. 

In both children and adults, the mean intervention effects became smaller after adjusting for confounding factors (table 2). The adjusted mean effect on salt intake was −0.25 g/day (−0.61 to 0.12) in children and −0.82 g/day (−1.24 to −0.40) in adults. The adjusted mean effect on blood pressure was −0.76 mm Hg (−2.37 to 0.86) for systolic blood pressure and −1.05 mm Hg (−2.45 to 0.34) for diastolic blood pressure in children and −1.64 mm Hg (−3.01 to −0.27) for systolic blood pressure and −0.48 mm Hg (−1.44 to 0.48) for diastolic blood pressure in adults (table 2).

Supplement table S2 shows the results from sensitivity analyses. The mean effect on salt intake was essentially unchanged when the analysis included possibly incomplete 24 hour urine collections or when the analysis only included the participants who completed the study with complete 24 hour urine collections, both at baseline and at the end of the trial. Supplement tables S3 and S4 show the post hoc subgroup results. No significant difference was seen between any of the subgroups in the intervention effect on salt intake or systolic blood pressure for both children and adults, except for the analysis of adults’ blood pressure status. The mean effect on salt intake was significantly greater in people with hypertension than in those with normal blood pressure (supplement table S3). The effect on systolic blood pressure showed a similar pattern, but the difference between individuals with hypertension and normal blood pressure was not statistically significant (supplement table S4).

## Discussion

Our study explores the use of mobile health technology to implement a salt reduction package including education, target setting, and self-monitoring, delivered to families via primary schools. In this multisite, cluster randomised controlled trial, the app based programme was feasible and effective in reducing salt intake and blood pressure in adults, but the effect on children’s salt intake was small and did not reach statistical significance. The reduction in salt intake of 0.82 g/day (that is, 8%) in adults might be considered modest; however, it represents an important decrease with public health significance, particularly given that the programme was carried out in a real world setting and could be easily scaled up. Based on a recent meta-analysis of blood pressure treatment trials,^22^ we estimated that the reduction of 1.64 mm Hg in systolic blood pressure observed in adults in our study would reduce the risk of stroke by 4.3% and ischaemic heart disease by 2.6%, which would prevent about 163 700 strokes and about 89 600 ischaemic heart disease events per year in China^23^ if the programme was scaled up across the country.

The lack of a significant effect of the intervention on children’s salt intake could be due to various factors. Firstly, about 60% of the children had school lunches regularly, but our intervention package did not include any school meal component except for salt awareness posters that were put up in canteens. Additionally, children might snack more than their parents,^24^ and most snacks are high in salt. Secondly, the app was installed on parents’ smartphones and children were only allowed access to them in the presence of their parents. As such, parents could have had more opportunities than children to gain knowledge or exchange experience with other participants. Some parents might have completed the AppSalt procedures on their own and, as a consequence, children might have received less education or were less involved in the salt reduction activities. Thirdly, some children might not have understood the online education messages to the same extent as their parents, despite all materials being designed for 8 to 9-year-old primary schoolchildren. Finally, our study sample size for children was small and did not have sufficient statistical power to detect a difference in salt intake of less than 1.5 g/day, whereas the intervention only resulted in a decrease of 0.3 g/day in salt intake in children.

Our results also showed a discrepancy in the mean salt intake and its changes over the 12 month intervention period between the two assessment methods—that is, 24 hour urinary sodium excretion and salt intake data collected from the app. The use of 24 hour urine collection is well known to be the most accurate method in assessing salt intake, whereas the dietary data recorded by the participants in the app, similar to all other dietary survey methods, is unreliable in estimating salt consumption^25^ because of various reasons, particularly inaccurate reporting or recording of the amount of salt and salty condiments used in cooking. Additionally, not all the salt recorded had been consumed—for example, some of the salt might have been used for soaking eggs and discarded in the liquid rather than ingested. Owing to the limitations of the estimated salt intake from the app, we have focused our report on the salt intake measured by 24 hour urinary sodium excretion. Nevertheless, the seven day salt estimation through the app provides a useful tool to keep the participants engaged in salt reduction and help them to reduce their salt intake.

Our post hoc analysis showed no significant difference in the intervention effect on salt intake among most subgroups, but individuals with raised blood pressure achieved a greater reduction in salt intake than those with normal blood pressure. This finding suggests that individuals are more likely to change their dietary habits to reduce salt intake, partially due to their high blood pressure. The post hoc subgroup results should be interpreted with caution because the sample size in each group was small.

### Comparison with other studies

The reduction in salt intake achieved in our current study was much smaller, for both children and adults, than that in the previous School-EduSalt programme where salt intake was reduced by 1.9 g/day in children and 2.9 g/day in adults.^14^ Although a direct comparison between the two studies is not possible, the difference in effect size is probably due to multiple factors. Firstly, the intervention duration in the current study is longer than that in School-EduSalt (one year *v* 3.5 months). Studies have shown that salt reduction achieved by dietary advice attenuates over time and maintaining a lower salt intake beyond several months remains a challenge.^2^ In our study, 24 hour urine collections were made at baseline and at 12 months only; thus, whether the intervention effect was larger at the early stage of the study is difficult to ascertain. The salt intake data estimated through the app every three to four months suggested a gradual and sustained decrease in salt intake during the 12 month intervention. However, as an intervention tool, such data were collected in the intervention group only, and therefore cannot be used to infer the outcome (that is, the difference between the two randomised groups). 

Secondly, the intervention in our current study was less intensive (nine lessons, 10 minutes each, over one year) than in School-EduSalt (eight lessons, 45 minutes each, over 3.5 months). Thirdly, children were slightly younger in the current study than those in School-EduSalt (8-9 years old in grade 3 *v* 10-11 years old in grade 4). Older children would have a better understanding and more effective communication skills, which could result in a greater intervention effect. We enrolled younger children in our current study mainly because of the longer duration of intervention and follow-up, and the recruitment of schoolchildren in early year education would ensure that the study could be completed before children became busier with their studies and examinations in the final year of primary school. Fourthly, in our current study, salt reduction education was delivered online via AppSalt, whereas the School-EduSalt lessons were delivered face to face. Although both learning methods have their advantages and limitations, our study was not designed to compare the two, and thus it is unclear whether the intervention effect was influenced by how lessons were delivered between these two studies.

The decrease of 1.64 mm Hg in systolic blood pressure for a reduction in salt intake of 0.82 g/day in adults observed in our study after adjusting for all potential confounding factors, is slightly bigger than that reported in the meta-analysis of salt reduction trials.^2^ This difference is most likely due to the longer duration of one year in our study than in most other trials included in the meta-analysis (with duration of only a few weeks); short term trials have been shown to underestimate the effect of salt reduction on blood pressure.^2^ The fall in systolic blood pressure that occurred with salt reduction in our study is close to that observed with a reduction in population salt intake over several years.^9^

### Strengths and limitations of this study

The strengths of our study included standardised field researchers’ training, a stringent and standardised protocol used across all study sites, high follow-up rates of 95% (565/592) for children and 91% (1072/1184) for adults, consistent use of electronic data collection system to ensure high quality data collection, two 24 hour urine collections at each time point to increase accuracy, and central laboratory measurement of urinary electrolytes without knowledge of whether the samples were from participants in the control or intervention group, which avoided potential bias on urinary sodium measurement. The use of automated blood pressure monitor minimised biases with respect to blood pressure measurements.

Our study also had some limitations, particularly a lack of intervention on school meals, which was probably the main reason for failing to achieve a significant reduction in salt intake in children. Another limitation was the reliance on smartphones, which was challenging for some grandparents, thus affecting the intervention implementation in those families. Similarly, this limitation would also affect the reach of the intervention to individuals who lived in remote rural areas with limited access to smartphones and internet.

### Public health implications

Our study findings provide further support to the notion that primary schools could act as a channel to promote the health of their families.^14^ This model has been used in several other studies to help parents stop smoking,^26^ improve cardiovascular disease risk score,^27^ increase physical activity, and lose weight.^28^ Given the high completion rate of primary education worldwide and the close parent-child relationship, school based intervention would be an efficient and valuable approach for cardiovascular disease prevention among family members.

This study also advances our understanding of how mobile health technology can be used in health education. With proper training and supervision, even individuals with minimal knowledge on the technology can learn to successfully use an app based decision support system to acquire knowledge, set goals, and monitor progress. Importantly, the app based education programme could substantially reduce the workload for schoolteachers and ease the pressure on the already busy school curriculum in China.

We carried out our study in three sites in northern, central, and southern China. Therefore, our findings should broadly apply to many primary schools in China and could potentially be adapted by other countries where most of the salt in the diet is added by the consumers. A wider scale implementation would have a major impact on reducing salt intake across populations and thereby preventing cardiovascular disease.

### Conclusions

Our study demonstrated that an app based education programme delivered through primary school, using a child-to-parent approach, was effective in lowering salt intake and blood pressure in children’s adult family members, although the effect on children’s salt intake was small and not significant. While this novel approach could potentially be scaled up to larger populations, a more comprehensive strategy, particularly with the inclusion of upstream policy interventions^29^ (eg, mandatory reformulation to improve the food environment), will be needed to effectively reduce salt intake across the whole population including children.

**What is already known on this topic**

Salt reduction lowers blood pressure and reduces the risk of cardiovascular disease
Salt intake in China is over twice the level recommended by the World Health Organization; most salt in the Chinese diet is added by the consumers themselves, which makes it difficult to limit
A previous study in northern China has demonstrated that an education programme targeted at primary school children (School-EduSalt) is effective in lowering salt intake in children and their families over one school term (about 3.5 months)

**What this study adds**

A smartphone application incorporating School-EduSalt (AppSalt) has been developed for children and their families to learn about salt reduction, estimate daily salt intake, set salt reduction targets, and monitor progress, to help them achieve and sustain lower salt intake in the long term
In a multisite, cluster randomised controlled trial in China, AppSalt was delivered through primary school using a child-to-parent approach; it was feasible and effective in reducing salt intake and systolic blood pressure in adults over one year, but the effects were not significant in children (owing to various factors such as a lack of intervention on school meals)
The app based education programme could potentially be scaled up across China, which could lead to a reduction in population salt intake and cardiovascular disease morbidity and mortality

## Data Availability

Relevant anonymised patient level data will be made available one year after publication of the primary manuscript on request from the corresponding author. Request for data sharing will be handled in line with the relevant regulations for data access and sharing in China and will need the approval of the trial steering committee, Peking University Institutional Review Board and Queen Mary (University of London) Ethics of Research Committee.

## Web extra

Extra material supplied by authors

Web appendix: Supplementary material

## Notes

Contributors: FJH and PZ conceived the project and contributed equally to the work. FJH, PZ, YLi, and RL designed the study and oversaw the conceptualisation and development of the app. PZ, YLi, RL, FC, YZ, WZ, DL, HC, TW, JY, CL, and SZ were responsible for setting up the study in each site. YLiu and RL led the development and maintenance of the app. XL, JS, MT, and LD performed statistical analyses. All authors contributed to the development of the intervention and evaluation. FJH wrote the first draft of the manuscript, and all authors contributed to the revision and approved the final manuscript. The corresponding author attests that all listed authors meet authorship criteria and that no others meeting the criteria have been omitted. FJH and PZ are the guarantors of this manuscript.

FJH and PZ affirm that the manuscript is an honest, accurate, and transparent account of the study being reported; that no important aspects of the study have been omitted; and that any discrepancies from the study as planned (and, if relevant, registered) have been explained.

Dissemination to participants and related patient and public communities: The results will be disseminated to schoolteachers, children, and their families through various channels, such as newsletters, WeChat, and the project website (https://www.actionsaltchina.com). We will discuss the results with participants, schoolteachers, policymakers, and other stakeholders on how to translate the research findings into practice. The results of this study will also be disseminated widely through national and international conference presentations, press release, and social media. Furthermore, the findings will be disseminated to a broader audience worldwide through World Action on Salt and Health, which is a global non-profit charitable organisation with 600 members from 100 countries with the mission to improve the health of populations by reducing salt intake globally.

Provenance and peer review: Not commissioned; externally peer reviewed.
