# Supplementary material for: App based education programme to reduce salt intake (AppSalt) in schoolchildren and their families in China: parallel, cluster randomised controlled trial
Source: BMJ. 2022 Feb 10;376:e066982. doi: 10.1136/bmj-2021-066982 (PMC8826455; doi:10.1136/bmj-2021-066982)
Supplement: Supplementary file 1 — Web appendix: Supplementary material [file hef066982.ww.pdf]

Supplementary Material

App based education programme to reduce salt intake (AppSalt) in schoolchildren and their families in China: parallel, cluster randomised controlled trial

Feng J He, Puhong Zhang, Rong Luo, Yuan Li, Yuewen Sun, Fengge Chen, Yuhong Zhao, Wei Zhao, Daoxi Li, Hang Chen, Tianyong Wu, Jianyun Yao, Changxing Lou, Siyuan Zhou, Le Dong, Yu Liu, Xian Li, Jing He, Changqiong Wang, Monique Tan, Jing Song, Graham A MacGregor

Table of Contents

Supplement 1. Description of the app based salt reduction intervention activities.

Table S1. Schedule of the intervention activities.

Figure S3. Changes in the average salt intake during the 12 month intervention period for children and adults in the intervention group (values were generated by the app using the seven day salt estimation method).

Table S2. Sensitivity analysis for salt intake (g/day) as calculated from 24 hour urinary sodium excretion**.**

Table S3. Salt intake (g/day) as measured by 24 hour urinary sodium excretion by subgroup.

Table S4. Systolic blood pressure (mm Hg) by subgroup.

Supplement 1: Description of the app based salt reduction intervention activities

The app based salt reduction education (AppSalt) is an m-Health intervention programme. A smartphone app named "AppSalt" was designed to provide a platform for delivering standardised education courses and tasks to grade 3 primary schoolchildren (8-9 years old) and their parents and grandparents. The app was installed in parents' or grandparents' smartphones and only adults were authorised to operate it. Children's tasks were to ensure that their parents or grandparents complete the lessons together with them and get the whole family involved in salt reduction activities. Besides the app, we implemented various offline activities. In total, there were five intervention components in the AppSalt programme, aiming to mobilise children to get their whole family to reduce salt intake. The details of the five intervention strategies of the AppSalt programme are described below.

1. Online education courses delivered through smartphone app

Over the 12 month intervention period, there was a total of 9 salt reduction education lessons (Table S1) and 12 usual health education lessons as normally included in the curriculum that did not contain salt-related material, e.g. flu prevention. All lessons were delivered through the app. A detailed description of the app including its features and functions has been published previously.[1] Figures S1 and S2 are two examples of the app (NB: The app is in Chinese and a few key words were translated into English and added to the screenshots).

Each lesson consisted of a 10-min video and a quiz to re-enforce important messages. Most of these lessons had a practical session to help participants put what they have learned into practice. The salt reduction education lessons covered knowledge and techniques for reducing salt intake, including the harmful effects of salt on health, the recommended level of salt intake, skills in reducing salt used in cooking and information on the sodium-reduced and potassium-enriched salt substitutes. Children and their family members could decide when and where to learn the lessons at their convenience.

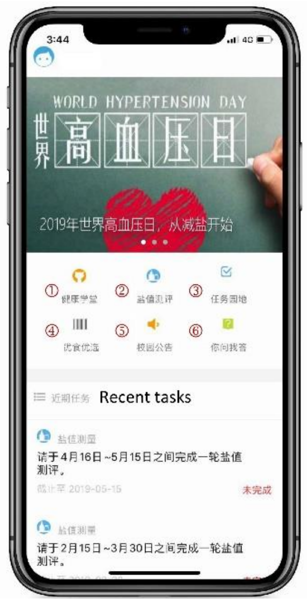

Figure S1. Screenshot of the app displaying the main functions: (1) Health education classroom; (2) Salt intake estimation; (3) Tasks; (4) Healthier food choices; (5) Notification board; (6) Questions and answers.

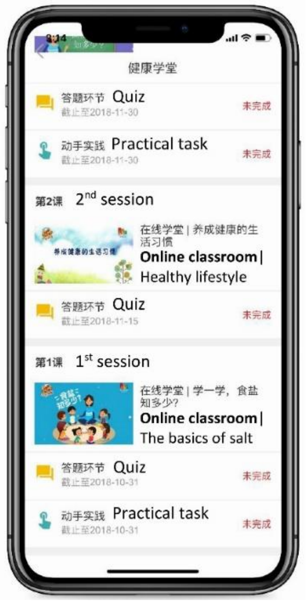

Figure S2. Screenshot of the "Health education classroom" in the app

2. Estimating and monitoring salt intake

The estimation and monitoring of salt intake was a task for the participants to complete and the data were recorded in the app. This task consisted of a seven day diary on salt intake for the family, which included weighing salt and other condiments (e.g. soy sauce) used in home cooking, estimating the consumption of processed food high in salt, and recording the frequency of eating out during the seven days. After completing this diary, the app calculated the average salt consumption using an embedded algorithm and generated a salt reduction action plan for each family member according to their salt intake and major sources of salt in their diet. All families were asked to complete this procedure at baseline and every three to four months thereafter. The information obtained could help each family member set a lower salt intake target and salt reduction targets for the top 3 contributors, e.g. reducing salt used in cooking by 50%. At the same time, the data shown on the app could help individuals monitor their progress on achieving lower salt intake.

3. Competitions and awards

A total of four competitions were organized by the schoolteachers (Table S1), including one art competition, two knowledge competitions, and one writing competition. After each competition, the top 30 children at each study site were awarded certificates and prizes.

4. Parent meetings

During the 12 month intervention period, schoolteachers were required to organise three to four face-to-face seminars for both children and adults to encourage peer communications and to collect their feedbacks on the programme. These seminars coincided with the schools' parent meetings, usually taken place at the beginning and the end of each school term. The topics of the seminars were related to the salt reduction courses or activities around that time.

5. Supportive environment

Posters were provided to the schools to help create a supportive environment for salt reduction on campus. The themes of these posters corresponded to the topics of the salt reduction education videos delivered through the app. Besides, some practical tools for reducing salt intake were provided to families, including salt-restriction spoons and salt awareness stickers.

Table S1. Schedule of the intervention activities

Month	Online salt reduction course	Salt intake estimation task	Competition and award	Parent group meeting	Supportive environment
1	The basics of salt			1st meeting	Throughout the whole programme
2	High-salt food in our diet	1st task			
3	Low sodium salt				
4	Myths about reducing salt intake			2nd meeting (optional)	
5	First re-cap	2nd task	Artwork competition		
6			First knowledge competition		
7	How to reduce salt used in cooking?			3rd meeting	
8	Salt and pre-packaged food	3rd task			
9	How to reduce salt when eating out?				
10	Second re-cap		Second knowledge competition		
11		4th task	Writing competition		
12				4th meeting	

Reference

1. He FJ, Zhang P, Luo R, et al. An Application-based programme to reinforce and maintain lower salt intake (AppSalt) in schoolchildren and their families in China. *BMJ Open* 2019;9:e027793.

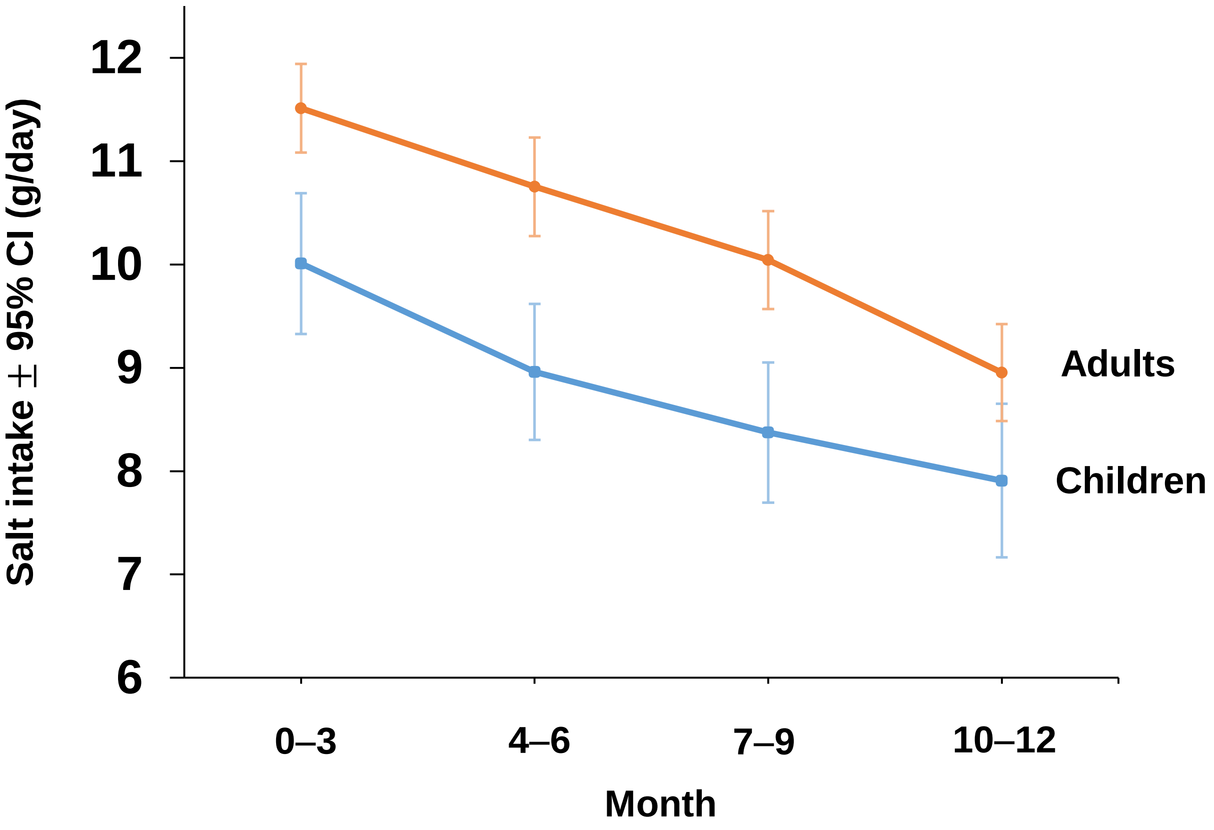

Figure S3. Changes in the average salt intake during the 12 month intervention period for children and adults in the intervention group (values were generated by the app using the seven day salt estimation method).

Table S2. Sensitivity analysis for salt intake (g/day) as calculated from 24 hour urinary sodium excretion

	Control				Intervention				Difference (95% CI) in change (intervention *v* control)†, P value		Adjusted Difference (95% CI) in change†‡ (intervention *v* control), P value	
	No of participants	Baseline mean (SD)	12 month mean (SD)	Change* (95% CI)	No of participants	Baseline mean (SD)	12 month mean (SD)	Change* (95% CI)				
**Including possibly incomplete 24 hour urine**												
Children	295	5.6 (2.1)	6.1 (2.3)	0.49 (0.24 to 0.75)	297	5.5 (2.0)	5.7 (2.3)	0.18 (-0.07 to 0.43)	-0.31 (-0.67 to 0.04)	0.09	-0.24 (-0.60 to 0.13)	0.20
Adults	590	10.0 (3.7)	9.8 (3.9)	-0.29 (-0.59 to 0)	594	10.0 (3.5)	8.9 (3.2)	-1.18 (-1.47 to -0.89)	-0.89 (-1.30 to -0.48)	<0.001	-0.82 (-1.24 to -0.40)	<0.001
**Including participants with complete 24 hour urine collections both at baseline and at the end of trial**												
Children	273	5.6 (2.1)	6.1 (2.3)	0.51 (0.25 to 0.77)	285	5.5 (1.9)	5.7 (2.3)	0.21 (-0.04 to 0.46)	-0.30 (-0.66 to 0.06)	0.11	-0.23 (-0.60 to 0.14)	0.22
Adults	516	10.1 (3.7)	9.8 (3.9)	-0.34 (-0.64 to -0.04)	541	10.0 (3.5)	8.9 (3.2)	-1.18 (-1.47 to -0.89)	-0.84 (-1.26 to -0.43)	<0.001	-0.77 (-1.20 to -0.35)	<0.001

SD=standard deviation.

*Comparison of the means between baseline and 12 month follow-up. Positive values=increases from baseline to 12 month follow-up; negative values=reductions from baseline to 12 month follow-up.

†Comparison between intervention and control groups in the changes from baseline to 12 month follow-up. Positive values=the intervention group had a greater increase or less decrease from baseline to 12 month follow-up than the control group; negative values=the intervention group has a greater decrease or smaller increase from baseline to 12 month follow-up than the control group.

‡Adjusted for age, sex, body mass index (body weight in children instead), outdoor temperature, study site, highest education level in the family.

Table S3. Salt intake (g/day) as measured by 24 hour urinary sodium excretion by subgroup

	Control				Intervention				Difference (95% CI) in change (intervention *v* control)†, P value		Adjusted Difference (95% CI) in change†‡ (intervention *v* control), P value		P for interaction‡
	No of participants	Baseline mean (SD)	12 month mean (SD)	Change* (95% CI)	No of participants	Baseline mean (SD)	12 month mean (SD)	Change* (95% CI)					
**Children**													
**Sex**													
Boys	156	6.3 (2.0)	6.5 (2.3)	0.18 (-0.17 to 0.53)	152	6.0 (2.1)	6.0 (2.5)	0.05 (-0.30 to 0.40)	-0.13 (-0.62 to 0.37)	0.62	-0.07 (-0.56 to 0.43)	0.79	0.27
Girls	138	4.8 (1.8)	5.7 (2.2)	0.91 (0.54 to 1.28)	144	5.0 (1.6)	5.3 (1.9)	0.33 (-0.03 to 0.69)	-0.58 (-1.10 to -0.07)	0.03	-0.47 (-0.98 to 0.05)	0.08	
**Site**													
Shijiazhuang	98	5.4 (2.1)	5.6 (2.5)	0.22 (-0.22 to 0.66)	98	5.6 (2.2)	5.7 (2.3)	0.12 (-0.32 to 0.56)	-0.10 (-0.72 to 0.52)	0.75	-0.19 (-0.80 to 0.42)	0.55	0.46
Yueyang	97	5.4 (2.0)	6.2 (2.3)	0.72 (0.26 to 1.18)	99	5.4 (1.6)	5.9 (2.1)	0.53 (0.09 to 0.96)	-0.20 (-0.83 to 0.44)	0.55	-0.05 (-0.70 to 0.59)	0.87	
Luzhou	99	5.9 (2.0)	6.5 (1.9)	0.64 (0.20 to 1.08)	99	5.5 (2.0)	5.4 (2.4)	-0.09 (-0.52 to 0.35)	-0.73 (-1.34 to -0.11)	0.02	-0.59 (-1.21 to 0.04)	0.07	
**Highest education in the family**													
Secondary school or lower	80	5.5 (2.0)	6.5 (2.6)	0.99 (0.48 to 1.49)	98	5.7 (2.1)	5.9 (2.4)	0.31 (-0.13 to 0.74)	-0.68 (-1.34 to -0.01)	0.05	-0.57 (-1.24 to 0.09)	0.09	0.60
High school	99	5.6 (1.9)	6.0 (2.3)	0.40 (-0.04 to 0.83)	92	5.7 (2.0)	5.9 (2.3)	0.15 (-0.30 to 0.60)	-0.25 (-0.87 to 0.38)	0.44	-0.18 (-0.79 to 0.44)	0.58	
University or college	115	5.6 (2.2)	5.9 (2.0)	0.32 (-0.10 to 0.73)	106	5.2 (1.7)	5.2 (2.0)	0.09 (-0.33 to 0.52)	-0.22 (-0.81 to 0.37)	0.46	-0.16 (-0.75 to 0.42)	0.58	
**Adults**													
**Age**													
<40	305	10.2 (3.6)	9.8 (3.6)	-0.55 (-0.96 to -0.15)	269	10.5 (3.5)	9.3 (3.2)	-1.22 (-1.65 to -0.80)	-0.67 (-1.26 to -0.08)	0.03	-0.63 (-1.22 to -0.04)	0.04	0.60
40-<60	155	9.9 (3.5)	9.9 (3.9)	0.07 (-0.50 to 0.64)	172	9.9 (3.6)	8.8 (3.4)	-1.13 (-1.65 to -0.60)	-1.20 (-1.97 to -0.42)	0.003	-1.11 (-1.90 to -0.32)	0.006	
⩾60	127	9.8 (3.8)	9.7 (4.5)	-0.15 (-0.77 to 0.47)	152	9.4 (3.4)	8.3 (2.9)	-1.17 (-1.74 to -0.60)	-1.02 (-1.86 to -0.18)	0.02	-0.96 (-1.81 to -0.12)	0.03	

	Control				Intervention				Difference (95% CI) in change (intervention *v* control)†, P value		Adjusted Difference (95% CI) in change†‡ (intervention *v* control), P value		P for interaction‡
	No of participants	Baseline mean (SD)	12 month mean (SD)	Change* (95% CI)	No of participants	Baseline mean (SD)	12 month mean (SD)	Change* (95% CI)					
**Sex**													
Male	277	10.6 (3.7)	10.8 (4.1)	0.05 (-0.38 to 0.47)	272	10.8 (3.8)	9.6 (3.4)	-1.13 (-1.56 to -0.71)	-1.18 (-1.78 to -0.58)	<0.001	-1.12 (-1.73 to -0.51)	<0.001	0.19
Female	310	9.5 (3.5)	9.0 (3.5)	-0.57 (-0.97 to -0.18)	321	9.4 (3.1)	8.2 (2.8)	-1.20 (-1.59 to -0.82)	-0.63 (-1.18 to -0.07)	0.03	-0.56 (-1.12 to 0)	0.05	
**Site**													
Shijiazhuang	198	10.9 (3.9)	9.9 (3.7)	-0.99 (-1.47 to -0.51)	198	10.8 (4.0)	9.3 (3.5)	-1.51 (-1.99 to -1.02)	-0.52 (-1.20 to 0.16)	0.14	-0.51 (-1.19 to 0.17)	0.15	0.11
Yueyang	193	9.3 (3.2)	9.6 (3.8)	0.10 (-0.42 to 0.63)	197	9.8 (3.3)	9.1 (3.0)	-0.61 (-1.11 to -0.12)	-0.71 (-1.43 to 0.01)	0.05	-0.77 (-1.51 to -0.03)	0.04	
Luzhou	196	9.9 (3.6)	9.9 (4.1)	0.06 (-0.44 to 0.56)	198	9.6 (3.1)	8.1 (2.9)	-1.41 (-1.91 to -0.92)	-1.47 (-2.18 to -0.77)	<0.001	-1.54 (-2.27 to -0.82)	<0.001	
**Education level**													
Secondary school or lower	245	10.3 (4.0)	10.1 (4.3)	-0.23 (-0.68 to 0.23)	278	10.0 (3.6)	8.7 (3.2)	-1.32 (-1.74 to -0.89)	-1.09 (-1.71 to -0.47)	<0.001	-1.10 (-1.73 to -0.46)	<0.001	0.12
High school	167	9.9 (3.6)	9.4 (3.7)	-0.53 (-1.07 to 0)	162	10.1 (3.2)	9.4 (3.0)	-0.73 (-1.27 to -0.20)	-0.20 (-0.96 to 0.56)	0.60	-0.16 (-0.92 to 0.60)	0.68	
University or college	175	9.8 (3.1)	9.7 (3.5)	-0.17 (-0.70 to 0.36)	153	10.1 (3.7)	8.6 (3.2)	-1.43 (-2.00 to -0.87)	-1.27 (-2.04 to -0.50)	0.001	-1.14 (-1.92 to -0.36)	0.004	
**Blood pressure status**													
Normotensive	453	9.9 (3.5)	9.5 (3.7)	-0.41 (-0.74 to -0.08)	455	9.8 (3.3)	8.8 (3.1)	-0.96 (-1.28 to -0.63)	-0.55 (-1.01 to -0.09)	0.02	-0.50 (-0.98 to -0.03)	0.04	0.004
Hypertensive§	134	10.7 (3.9)	10.9 (4.4)	0.11 (-0.51 to 0.73)	138	10.9 (4.1)	9.0 (3.4)	-1.90 (-2.49 to -1.31)	-2.01 (-2.86 to -1.16)	<0.001	-1.95 (-2.81 to -1.09)	<0.001	
**Adults' relationship with children**													
Parents	192	10.5 (3.3)	10.6 (3.7)	-0.08 (-0.60 to 0.43)	180	11.0 (3.9)	9.7 (3.5)	-1.31 (-1.84 to -0.79)	-1.23 (-1.96 to -0.49)	0.001	-1.20 (-1.94 to -0.46)	0.002	0.41
Grandparents	260	9.8 (3.8)	9.5 (3.9)	-0.43 (-0.86 to 0.01)	250	9.8 (3.1)	8.7 (2.9)	-1.07 (-1.50 to -0.63)	-0.64 (-1.25 to -0.03)	0.04	-0.54 (-1.16 to 0.08)	0.09	

	Control				Intervention				Difference (95% CI) in change (intervention *v* control)†, P value		Adjusted Difference (95% CI) in change†‡ (intervention *v* control), P value		P for interaction‡
	No of participants	Baseline mean (SD)	12 month mean (SD)	Change* (95% CI)	No of participants	Baseline mean (SD)	12 month mean (SD)	Change* (95% CI)					
Others	135	9.7 (3.7)	9.3 (3.9)	-0.33 (-0.94 to 0.28)	163	9.4 (3.4)	8.2 (3.0)	-1.19 (-1.74 to -0.64)	-0.86 (-1.68 to -0.04)	0.04	-0.83 (-1.65 to -0.01)	0.05	

SD=standard deviation.

*Comparison of the means between baseline and 12 month follow-up. Positive values=increases from baseline to 12 month follow-up; negative values=reductions from baseline to 12 month follow-up.

†Comparison between intervention and control groups in the changes from baseline to 12 month follow-up. Positive values=the intervention group had a greater increase or less decrease from baseline to 12 month follow-up than the control group; negative values=the intervention group has a greater decrease or smaller increase from baseline to 12 month follow-up than the control group.

‡Adjusted for age, sex, body mass index (body weight in children instead), outdoor temperature, study site, highest education level in the family.

§ Defined as systolic blood pressure ≥140 mm Hg or diastolic blood pressure ≥90 mm Hg or self-reported hypertension.

Table S4. Systolic blood pressure (mm Hg) by subgroup

	Control				Intervention				Difference (95% CI) in change (intervention *v* control)†, P value		Adjusted Difference (95% CI) in change†‡ (intervention *v* control), P value		P for interaction‡
	No of participants	Baseline mean (SD)	12 month mean (SD)	Change* (95% CI)	No of participants	Baseline mean (SD)	12 month mean (SD)	Change* (95% CI)					
**Children**													
**Sex**													
Boys	156	93.8 (9.5)	96.5 (7.8)	2.48 (0.94 to 4.02)	152	94.0 (9.4)	96.0 (8.9)	1.98 (0.43 to 3.53)	-0.50 (-2.68 to 1.67)	0.65	-0.01 (-2.22 to 2.20)	0.99	0.33
Girls	139	91.0 (10.0)	93.9 (9.4)	2.85 (1.22 to 4.48)	145	91.6 (9.8)	92.6 (10.6)	0.95 (-0.62 to 2.52)	-1.90 (-4.16 to 0.36)	0.10	-1.57 (-3.87 to 0.72)	0.18	
**Site**													
Shijiazhuang	99	94.7 (9.9)	96.3 (8.6)	1.58 (-0.31 to 3.47)	99	96.2 (9.7)	96.4 (10.8)	0.17 (-1.73 to 2.08)	-1.41 (-4.08 to 1.27)	0.30	-1.80 (-4.47 to 0.88)	0.19	0.56
Yueyang	97	90.4 (9.2)	94.4 (8.3)	4.12 (2.12 to 6.12)	99	90.5 (9.5)	92.9 (8.9)	2.34 (0.43 to 4.25)	-1.77 (-4.53 to 0.99)	0.21	-1.25 (-4.09 to 1.59)	0.39	
Luzhou	99	92.4 (9.9)	95.0 (9.1)	2.35 (0.44 to 4.26)	99	91.9 (9.0)	93.6 (9.7)	1.86 (-0.04 to 3.77)	-0.48 (-3.18 to 2.21)	0.73	0.25 (-2.50 to 2.99)	0.86	
**Highest education in the family**													
Secondary school or lower	80	92.1 (10.9)	95.8 (8.8)	3.42 (1.24 to 5.61)	98	94.0 (9.5)	95.5 (9.4)	1.63 (-0.28 to 3.54)	-1.79 (-4.69 to 1.11)	0.23	-1.38 (-4.35 to 1.59)	0.36	0.44
High school	100	93.5 (9.4)	96.6 (8.1)	3.06 (1.18 to 4.95)	93	93.2 (10.0)	94.2 (9.3)	0.89 (-1.09 to 2.86)	-2.18 (-4.90 to 0.55)	0.12	-1.84 (-4.58 to 0.91)	0.19	
University or college	115	91.9 (9.4)	93.6 (9.0)	1.72 (-0.08 to 3.52)	106	91.5 (9.5)	93.4 (10.9)	1.79 (-0.07 to 3.64)	0.07 (-2.51 to 2.65)	0.96	0.49 (-2.12 to 3.09)	0.72	
**Adults**													
**Age**													
<40	306	112.0 (13.6)	112.5 (14.8)	0.71 (-0.63 to 2.05)	269	111.7 (14.9)	109.8 (14.3)	-1.39 (-2.80 to 0.02)	-2.10 (-4.05 to -0.16)	0.03	-1.45 (-3.37 to 0.47)	0.14	0.91
40-<60	157	122.5 (14.8)	121.2 (15.5)	-0.99 (-2.88 to 0.90)	173	120.9 (16.4)	117.2 (15.8)	-4.20 (-5.94 to -2.45)	-3.20 (-5.77 to -0.63)	0.02	-2.02 (-4.57 to 0.53)	0.12	
⩾60	127	131.2 (17.7)	129.2 (19.8)	-2.06 (-4.11 to -0.01)	152	128.7 (17.8)	124.5 (17.0)	-3.91 (-5.80 to -2.02)	-1.85 (-4.63 to 0.94)	0.19	-1.25 (-3.99 to 1.49)	0.37	

	Control				Intervention				Difference (95% CI) in change (intervention *v* control)†, P value		Adjusted Difference (95% CI) in change†‡ (intervention *v* control), P value		P for interaction‡
	No of participants	Baseline mean (SD)	12 month mean (SD)	Change* (95% CI)	No of participants	Baseline mean (SD)	12 month mean (SD)	Change* (95% CI)					
**Sex**													
Male	279	124.9 (15.4)	124.8 (16.1)	-0.23 (-1.65 to 1.20)	272	123.0 (15.6)	120.7 (15.7)	-2.25 (-3.67 to -0.83)	-2.02 (-4.04 to -0.01)	0.05	-1.04 (-3.03 to 0.95)	0.31	0.42
Female	311	113.5 (16.2)	113.1 (16.9)	-0.36 (-1.69 to 0.96)	322	115.1 (18.4)	111.6 (16.1)	-3.34 (-4.63 to -2.05)	-2.98 (-4.82 to -1.13)	0.002	-2.13 (-3.95 to -0.30)	0.02	
**Site**													
Shijiazhuang	198	119.1 (18.0)	118.5 (17.6)	-0.36 (-1.95 to 1.23)	198	120.7 (17.6)	117.9 (16.5)	-2.57 (-4.19 to -0.96)	-2.21 (-4.47 to 0.05)	0.06	-2.17 (-4.38 to 0.05)	0.06	0.21
Yueyang	194	117.5 (15.9)	117.9 (17.1)	1.03 (-0.73 to 2.78)	198	116.1 (16.8)	115.3 (16.8)	-0.70 (-2.35 to 0.95)	-1.73 (-4.14 to 0.68)	0.16	0.07 (-2.36 to 2.50)	0.96	
Luzhou	198	120.2 (16.4)	118.8 (18.0)	-1.53 (-3.21 to 0.15)	198	119.4 (18.1)	113.8 (16.1)	-5.34 (-6.99 to -3.68)	-3.81 (-6.17 to -1.45)	0.002	-2.77 (-5.12 to -0.41)	0.02	
**Education level**													
Secondary school or lower	246	122.9 (17.0)	121.2 (18.0)	-1.47 (-2.99 to 0.05)	278	122.6 (18.3)	118.0 (16.5)	-4.44 (-5.84 to -3.04)	-2.97 (-5.04 to -0.91)	0.005	-2.25 (-4.31 to -0.20)	0.03	0.59
High school	169	117.9 (17.5)	118.2 (17.8)	0.10 (-1.67 to 1.87)	163	117.1 (16.3)	115.1 (16.8)	-2.01 (-3.80 to -0.22)	-2.11 (-4.62 to 0.40)	0.10	-1.36 (-3.83 to 1.11)	0.28	
University or college	175	114.3 (14.3)	115.0 (16.0)	0.85 (-0.93 to 2.62)	153	113.5 (16.1)	112.2 (15.6)	-0.95 (-2.82 to 0.92)	-1.79 (-4.37 to 0.78)	0.17	-0.58 (-3.11 to 1.95)	0.65	
**Blood pressure status**													
Normotensive	456	112.8 (11.5)	113.4 (13.1)	0.53 (-0.55 to 1.61)	456	112.2 (12.0)	110.5 (13.0)	-1.54 (-2.61 to -0.47)	-2.07 (-3.58 to -0.55)	0.008	-1.29 (-2.80 to 0.23)	0.10	0.15
Hypertensive§	134	139.6 (15.3)	136.6 (19.4)	-3.13 (-5.16 to -1.10)	138	140.4 (15.6)	132.9 (15.1)	-7.32 (-9.26 to -5.38)	-4.19 (-6.99 to -1.39)	0.003	-3.56 (-6.32 to -0.80)	0.01	

	Control				Intervention				Difference (95% CI) in change (intervention *v* control)†, P value		Adjusted Difference (95% CI) in change†‡ (intervention *v* control), P value		P for interaction‡
	No of participants	Baseline mean (SD)	12 month mean (SD)	Change* (95% CI)	No of participants	Baseline mean (SD)	12 month mean (SD)	Change* (95% CI)					
**Adults' relationship with children**													
Parents	194	121.8 (12.8)	121.9 (13.3)	0.41 (-1.30 to 2.12)	180	120.5 (14.0)	119.4 (13.8)	-1.06 (-2.80 to 0.68)	-1.46 (-3.90 to 0.97)	0.24	-0.65 (-3.05 to 1.75)	0.60	0.44
Grandparents	260	112.8 (16.6)	112.6 (17.2)	-0.04 (-1.48 to 1.40)	251	111.7 (16.5)	109.0 (15.7)	-2.57 (-4.02 to -1.12)	-2.53 (-4.57 to -0.49)	0.02	-1.48 (-3.50 to 0.54)	0.15	
Others	136	126.5 (18.0)	125.2 (19.6)	-1.80 (-3.81 to 0.22)	163	127.5 (18.4)	122.2 (16.9)	-5.21 (-7.03 to -3.40)	-3.41 (-6.12 to -0.71)	0.01	-2.95 (-5.61 to -0.29)	0.03	

SD=standard deviation.

*Comparison of the means between baseline and 12 month follow-up. Positive values=increases from baseline to 12 month follow-up; negative values=reductions from baseline to 12 month follow-up.

†Comparison between intervention and control groups in the changes from baseline to 12 month follow-up. Positive values=the intervention group had a greater increase or less decrease from baseline to 12 month follow-up than the control group; negative values=the intervention group has a greater decrease or smaller increase from baseline to 12 month follow-up than the control group.

‡Adjusted for age, sex, body mass index (body weight in children instead), outdoor temperature, study site, highest education level in the family, physical activity and alcohol consumption (adults only).

§Defined as systolic blood pressure ⩾140 mmHg or diastolic blood pressure ⩾90 mmHg or self-reported hypertension.
